# Elaboration and Characterization of Natural Deep Eutectic Solvents (NADESs): Application in the Extraction of Phenolic Compounds from *pitaya*

**DOI:** 10.3390/molecules27238310

**Published:** 2022-11-29

**Authors:** Ianê Valente Pires, Yasmin Caroline Nóvoa Sakurai, Nelson Rosa Ferreira, Sanclayton Geraldo Carneiro Moreira, Antonio Manoel da Cruz Rodrigues, Luiza Helena Meller da Silva

**Affiliations:** 1 Programa de Pós-Graduação em Ciência e Tecnologia de Alimentos, Postgraduate Program in Food Science and Technology, Universidade Federal do Pará, Rua Augusto Corrêa S/N, Belém 66075-900, PA, Brazil; 2 Instituto de Ciências Exatas e Naturais (ICEN), Universidade Federal do Pará, Rua Augusto Corrêa S/N, Belém 66075-900, PA, Brazil

**Keywords:** natural deep eutectic solvents, extraction, phenolic compounds, dragon fruit, Raman spectroscopy, FTIR

## Abstract

In this paper, natural deep eutectic solvents (NADESs) with lactic acid, glycine, ammonium acetate, sodium acetate, and choline chloride were prepared with and without the addition of water. NADES formation was evaluated using FTIR and Raman, where hydrogen bonds were identified between the hydroxyl group of lactic acid and the amino and carboxyl groups of glycine. Acetate and ammonium ions were also identified as forming bonds with lactic acid. The addition of water did not cause changes in the vibrational modes of the FTIR and Raman spectra but contributed to a reduction in NADES viscosity and density. Viscosity ranged from 0.335 to 0.017 Pa s^−1^, and density ranged from 1.159 to 0.785 g mL^−1^. The best results for the extraction of phenolic compounds from *pitaya* (dragon fruit) were achieved with an organic solvent (450. 41 mg 100 g^−1^ dry bases-db) in comparison to NADESs lactic acid:glycine (193.18 mg 100 g^−1^ db) and lactic acid:ammonium acetate (186.08 mg 100 g^−1^ db). The antioxidant activity of the extracts obtained with the NADESs was not statistically different from that of the extract obtained with organic solvents.

## 1. Introduction

Conventional organic solvents are widely used for the extraction of bioactive components in chemical, pharmaceutical, food, and cosmetic industries. However, most of them are characterized as harmful, both to health and the environment, due to their toxicity, flammability, and high volatility, not meeting the principles of green chemistry, which prioritizes substances having less impact on humans and the environment [1].

Natural deep eutectic solvents (NADESs) have important characteristics, such as sustainability, biodegradability, low volatility at room temperature, a high solubilization capacity, and selectivity, making NADESs a promising alternative to be used in the extraction of nutraceuticals, as well as to obtain food extracts [2,3,4,5,6].

NADES is a general term used to cover a wide range of primary metabolite compositions, which, in different combinations, form liquids at room temperature. NADES is obtained by mixing two or more natural metabolites in a specific molar ratio, forming a liquid due to chemical interactions and reducing their individual melting points [7].

This feature allows NADESs to be “tailored” by mixing two natural metabolites compatible with the chemical nature of the analyte to be extracted [8]. Several studies have focused on the analysis of the physicochemical properties of different NADES with the objective of identifying the most suitable mixture for each extraction process [9,10].

The main natural hydrogen-bond donor metabolite used for the preparation of NADESs is choline chloride due to its ability to bind most primary metabolites, in addition to glycerol and carboxylic acids. Regarding the molar proportions studied, over 95% of them used are between 1:1 and 1:4, with 44% of this total using the 1:1 proportion [11,12,13,14].

The formation of NADESs can be evaluated by different types of analyses, such as thermal differential scanning calorimetry (DSC), physical (viscosity and density), conductivity, and spectroscopic analyses, [15,16,17], with emphasis on viscosity, since this is an important property that helps make their industrial application viable [2].

The use of NADES as solvents in the extraction processes of bioactive compounds (total phenolic compounds, flavonoids, and anthocyanins) in different plant matrices has shown results similar to those when using conventional organic solvents [10,14,18,19].

These are promising results, since a greater stability of the bioactive compounds in the extracts is shown, which also allows for their direct application in the preparation of foods, pharmaceuticals, and cosmetics, giving them a great advantage over extracts obtained conventionally [20,21,22,23].

Ultrasound-assisted extraction (UAE) can be performed in seconds or minutes with high reproducibility, greater purity of the final product, simpler handling, reduced solvent consumption, and, consequently, reduced operating costs. It consumes only a fraction of the time and energy normally required in conventional processes, being considered a green technology. Other advantages of using UAE include more effective mixtures and micro-mixtures, faster energy and mass transfer, reduced thermal and concentration gradients, and reduced temperature and selective extraction. These factors enable the efficient use of UAE in the extraction of a wide class of compounds, such as essential oils, aromas, pigments, antioxidants, and other organic compounds [24]. The use of UAE combined with green solvents seems advantageous, as it not only improves yield and reduces the reaction times, but it also reduces energy consumption [25].

The consumption of exotic fruits has grown in recent decades due to claims of their properties in the prevention of cardiovascular diseases [26]. *Pitaya*, known as “dragon fruit” and belonging to the *Cactaceae* family, is native to the tropical regions of Mexico and Central and South America [27], being mainly distributed in tropical and subtropical regions [28]. Since it is rich in bioactive compounds, it has been the focus of several studies on extraction processes with conventional solvents [29,30,31,32,33].

Phenolic compounds found both in the pulp and in the peel of different dragon fruit species are not only used as food supplements but also as additives in medicine, cosmetics, and the food industry in order to increase the bioavailability of the product, prolonging its shelf-life [34,35,36].

This work aimed to elaborate and characterize NADESs formed using a mixture of lactic acid: glycine, lactic acid: ammonium acetate, lactic acid: sodium acetate, and lactic acid: choline chloride in different molar proportions and with the addition of water, as well as to test them as a solvent in the extraction of phenolic compounds from dragon fruit (*Hyloreceus Costaricensis*) pulp.

## 2. Results and Discussion

### 2.1. NADES Characterization

#### 2.1.1. FTIR

**Figure 1:**
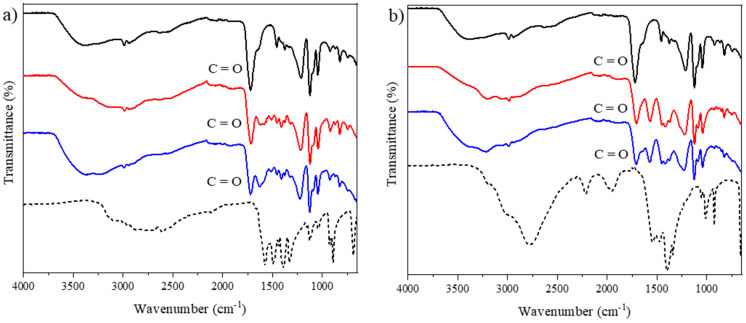
(**a**) FTIR spectra of — (black line) lactic acid, ---- (dashed line) glycine, **—** (red line) lactic acid:glycine, **—** (blue line) lactic acid:glycine:water. (**b**) FTIR spectra of — (black line) lactic acid, --- (dashed line) ammonium acetate, **—** (red line) lactic acid: ammonium acetate, **—** (blue line) lactic acid:ammonium acetate:water.

FTIR analysis was used to verify the formation of hydrogen bonds in the developed NADEs. Spectra of pure hydrogen donors and acceptors, as well as the developed NADESs, were analyzed. Figure 1 illustrates the spectra obtained for the lactic acid, the glycine, and the NADESs lactic acid:glycine and lactic acid:glycine:water.

The results confirm that changes occurred between the spectra of the pure metabolites and the NADESs in the range from 1210 to 1668 cm^−1^, a region where the main vibrational modes for lactic acid are found, such as the asymmetric bending of CH_3_ at 1457 cm^−1^ and the symmetrical folding of CH_3_ at 1378 cm^−1^. Furthermore, a peak characteristic of the presence of carboxylic acid stretching vibrations (C=O) was observed at 1720 cm^−1^ [37]. In the spectra of the pure compounds, it was possible to detect characteristic peaks of torsion of the group NH_3_^+^ (2602 cm^−1^) for glycine (Figure 1a), an elongation of CH (2779 cm^−1^) for ammonium acetate (Figure 1b), a stretching of C=O (1565 cm^−1^) for sodium acetate (Figure S3), and an elongation of OH (3215 cm^−1^) for choline chloride (Figure S4) [38,39,40].

In the NADESs spectra (Figure 1a), it was possible to verify the disappearance of the glycine peak corresponding to the symmetrical stretching of the group CCN (893 cm^−1^) [38], which can be attributed to the destruction of the crystal structure of the pure components due to the occurrence of the melting process. A similar behavior was observed in the NADESs formed using choline chloride and glycerol (1:3) [41].

In the spectra of the pure substances in relation to the NADESs spectra without the addition of water (red line), changes were observed in a wide range of vibrational bands (3060 to 3700 cm^−1^), which correspond to the absorption peak of the stretching vibration of the hydroxyl group (OH). This region has already been identified and attributed to the existence of multiple forms of hydrogen bonds for different NADESs by several researchers [17,37,42]. However, this region is strongly influenced by water molecules. The spectra of the FTIR, pure substances, and mixtures allow us to infer the formation of intermolecular interactions. This observation was also reported in other studies with NADESs [43].

**Figure 2:**
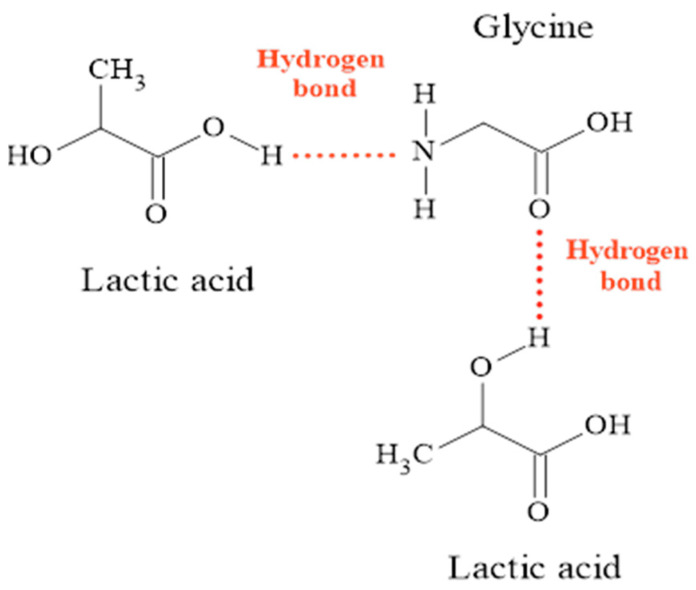
Preferential interactions between lactic acid and glycine.

**Figure 3:**
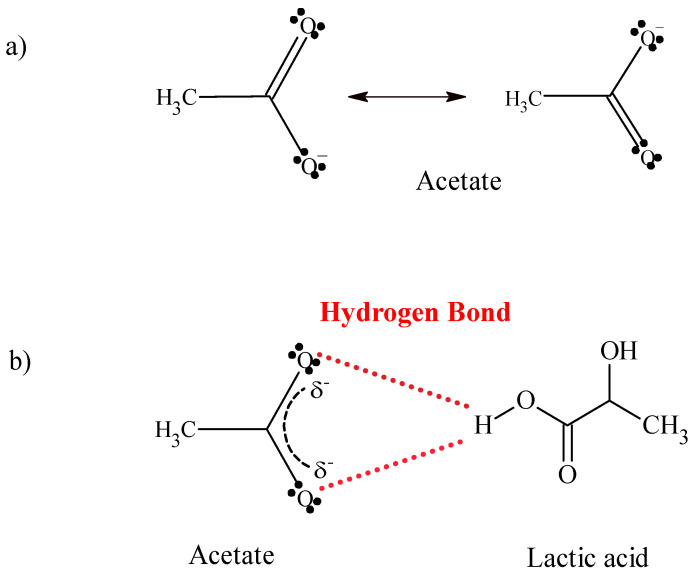
(**a**) Resonance stabilization of acetate ion. (**b**) Preferential interactions between lactic acid and acetate ion.

Hydrogen bonds can occur in different ways [44,45]. By analyzing the molecular structure of lactic acid and glycine individually, it can be assumed that, when the compounds are mixed, hydrogen interactions occur between the O-H group of lactic acid and the N-H or C=O group of glycine, forming bonds, such as O-H···N-H and O-H···C=O (Figure 2). When we explore the structure of ammonium acetate, another form of interaction with lactic acid can be hypothesized, taking into account the hybrid form of acetate ion (Figure 3a) and thus assuming hydrogen bonds between the O-H group in lactic acid and the C=O group in the acetate ion (Figure 3b).

The effect of adding water (blue lines) was evidenced by the increased intensity of the region corresponding to the OH group, which may indicate an increase in solvent polarity. However, when compared to the spectra of the NADESs without the addition of water, no major changes were observed in other vibrational modes of the molecules, indicating that the amount of water added did not interfere with NADES formation. It should be taken into account that each added water molecule can form up to four H bonds (two donors and two acceptors) [46]. 

**Figure 4:**
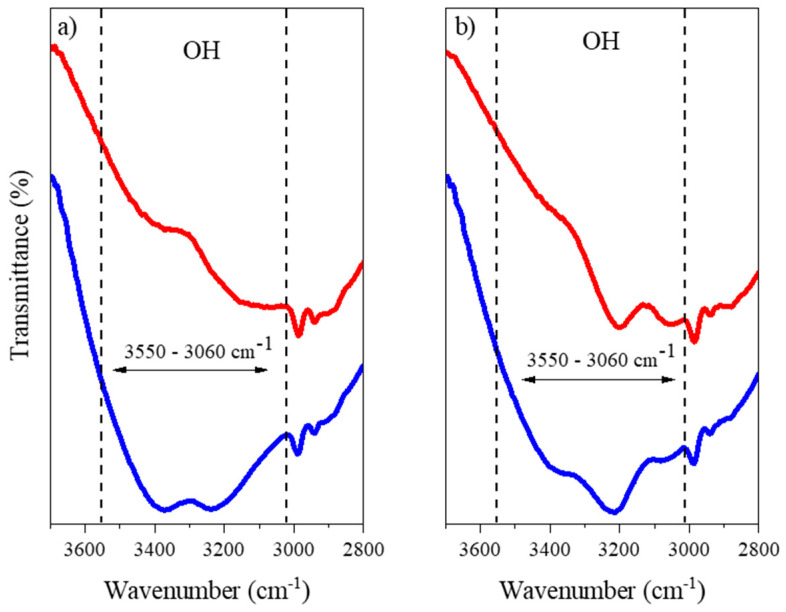
FTIR spectra characterizing O–H stretching. (**a**) FTIR spectra of **—** (red line) lactic acid:glycine, **—** (blue line) lactic acid:glycine:water. (**b**) FTIR spectra of **—** (red line) lactic acid: ammonium acetate, **—** (blue line) lactic acid:ammonium acetate:water.

Figure 4 shows a greater emphasis on the band corresponding to the regions of OH vibration, comparing the NADESs without the addition of water and those with the addition of water. It is possible to observe that there was no horizontal displacement between the samples, which is associated with a weakening of hydrogen bonds. Therefore, the addition of water in these proportions does not seem to weaken the hydrogen bonds present in these NADESs. A similar behavior in other NADESs analyzed using FTIR has already been reported [47].

**Figure 5:**
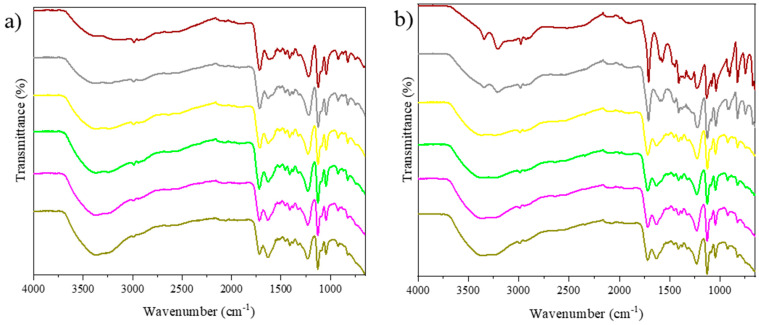
Spectra (FTIR) of lactic acid:glycine mixtures with different proportions of water. (**a**) One day after NADES preparation **—** (brown line) (5%); **—** (gris line) (10%); **—**(yellow line) (20%); **— **(green line) (30%); **—** (pink line) (40%); **—** (moss green line) (50%). (**b**) Eight months after NADES preparation **—** (brown line) (5%); **—** (gris line) (10%); **—** (yellow line) (20%); **— ** (green line) (30%); **—**(pink line) (40%); **—** (moss green line) (50%).

In order to deepen the effect of adding water to the mixtures, different proportions (5 to 50% water) were added to the NADESs lactic acid:glycine. The FTIR analyses were performed one day after the production of the NADESs and after eight months of storage in glass without the incidence of light so that the stability of the mixtures could also be evaluated (Figure 5).

According to the FTIR spectrum obtained (Figure 5a), the most evident change was the increased intensity of the region corresponding to OH as the percentage of water was increased, with no changes in other vibrational modes. However, after eight months (Figure 5b), the mixtures with 5 and 10% of water showed new peaks (3346 and 3208 cm^−1^, and 3344 and 3210 cm^−1^, respectively) in the OH region, probably due to the crystallization process of the solid residues of glycine. Since glycine is not completely solubilized in lactic acid in these proportions, these mixtures are unfeasible to be used as solvents.

FTIR proved to be capable of quickly and accurately reproducing the alteration of structures that occurred in the mixtures, with sufficient sensitivity to evaluate the formation of hydrogen bonds [48] and the consequent formation of the NADESs. It is important to emphasize that this technique has been frequently and successfully used for the characterization of solvents in green chemistry [41,49,50]. Figures S1–S4 in the Supplementary Materials show FTIR spectral graphics of individual components and the formed NADESs.

#### 2.1.2. Raman

**Figure 6:**
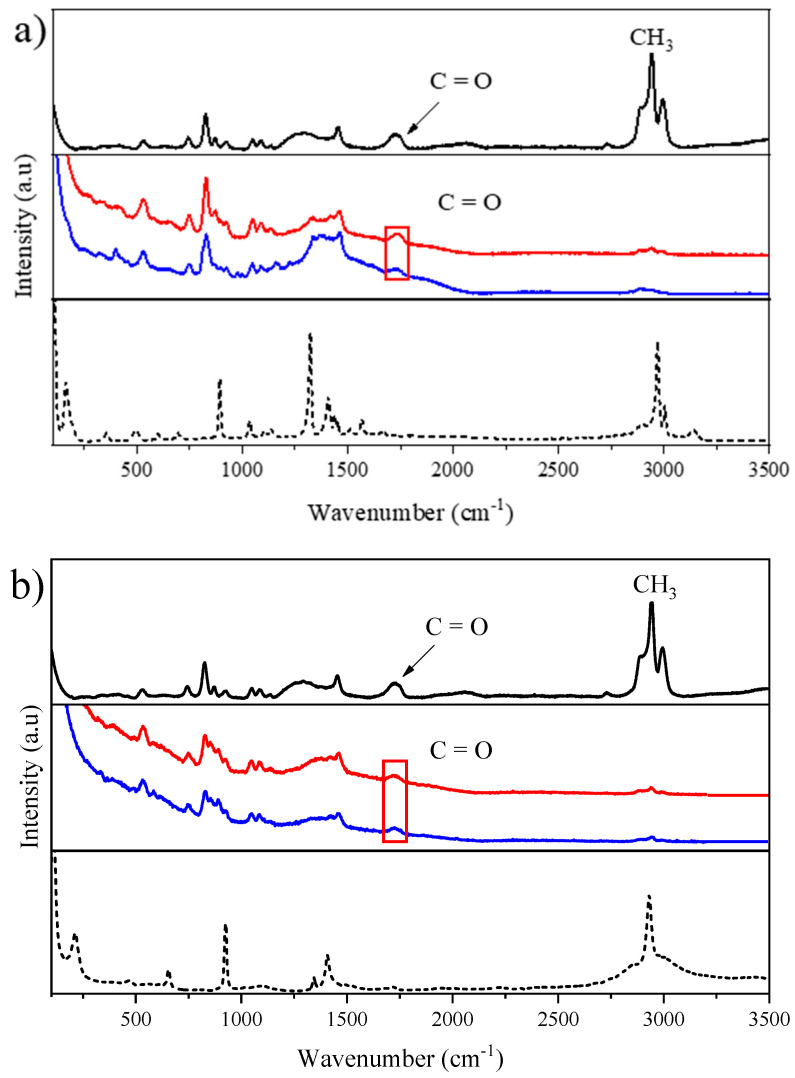
(**a**) Spectra (FTIR) of the pure components ***—*** (black line) lactic acid, ---- (dashed line) glycine, ***—*** (red line) NADESs without water; ***—*** (blue line) NADESs with water. (**b**) Raman spectra of the pure components ***—*** (black line) lactic acid lactic acid, ---- (dashed line) ammonium acetate, ***—*** (red line) NADESs without water; **—** (blue line) NADESs with water.

The same samples evaluated in FTIR were analyzed using the Raman technique, both with pure components and mixtures. The spectra corresponding to the region from 100 to 3500 cm^−1^ of lactic acid and the glycine mixture, as well as their precursors, are shown in Figure 6 and in Supplementary Materials (Figures S5–S8). 

Characteristic bands were identified in the spectrum of the pure lactic acid in the region between 2834 cm^−1^ and 3051 cm^−1^, referring to the different vibration modes of CH_3_ groups and the C=O group (stretching) in 1730 cm^−1^ [51]. An intense torsion band of the cluster was also detected for NH_3_^+^ (1323 cm^−1^), which is typical of pure glycine and other less intense bands, such as the elongation of C-C (893 cm^−1^) and CCN (1037 cm^−1^) groups [52,53].

Representative bands of higher intensity for the other pure compounds were identified (Supplementary Materials), such as the stretching of CH_3_ (2931 cm^−1^) of ammonium acetate, the elongation of C-C (929 cm^−1^) of sodium acetate, and the elongation of C-N (721 cm^−1^) of choline chloride [54,55]. 

By evaluating the spectra obtained from the pure components and mixtures, it was observed that changes occurred mainly due to the formation of hydrogen bonds, which indicate the occurrence of interactions between the pure components generating the NADESs. Unlike FTIR in Raman, the lower intensity of the vibrations of the O-H group indicated the most intense bands of lactic acid, referring to the different vibrations of CH_3_ (2800 cm^−1^ to 3000 cm^−1^), which disappear in the spectra of the mixtures, while the bands of the C=O group (stretching) at 1730 cm^−1^ appear in all the NADESs, having the same behavior found in the FTIR analysis spectra. This demonstrates the complementarity of the two vibrational techniques [56]. The disappearance and maintenance of the characteristic peaks in the mixtures when compared with those of the pure components emphasize the theory of the possible formation of NADESs.

The lactic acid:ammonium acetate and lactic acid:sodium acetate mixtures showed similar spectra due to the chemical structures of the two pure substances linked to lactic acid (acetates).

The Raman spectra of the NADESs with the addition of water practically did not change when compared to those without the addition of water, indicating that the added water did not interfere with the vibrational modes of the molecules. This fact can help to minimize the disadvantages caused by the high viscosity of NADESs in analytical and industrial processes.

Raman spectroscopy has been successfully used as an analytical technique for the identification of several alternative green solvents, such as ionic liquids and Deep Eutectic Solvents (DESs) [45,57,58]. The results found in this work with the Raman technique demonstrate success in proving the interaction between the precursor components of NADESs. Thus, this is a promising technique capable of providing information regarding the chemical and structural changes in substances in a simple and fast way using a small amount of sample.

#### 2.1.3. Rheological Behavior and Density

**Figure 7:**
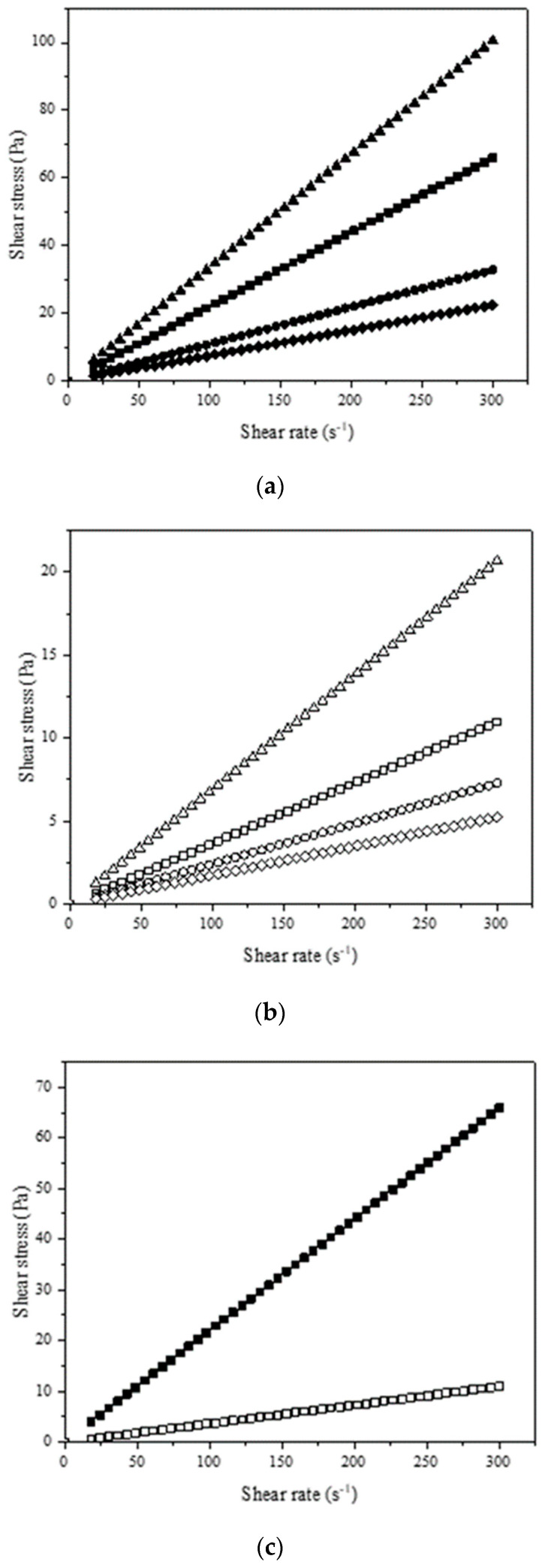
Flow curves for NADESs fitted to Newton model at 40 °C. (**a**) Solid symbols represent NADESs without the addition of water (■ lactic acid:glycine; ● lactic acid:ammonium acetate; ▲ lactic acid:ammonium acetate; ♦ lactic acid:choline chloride); (**b**) hollowed out symbols represent NADESs with the addition of water (□ lactic acid:glycine; ○ lactic acid:ammonium acetate; Δ lactic acid: sodium acetate; ◊ lactic acid:choline chloride). (**c**) Comparison of NADESs behavior with and without the addition of water (lactic acid:glycine ■; □).

**Figure 8:**
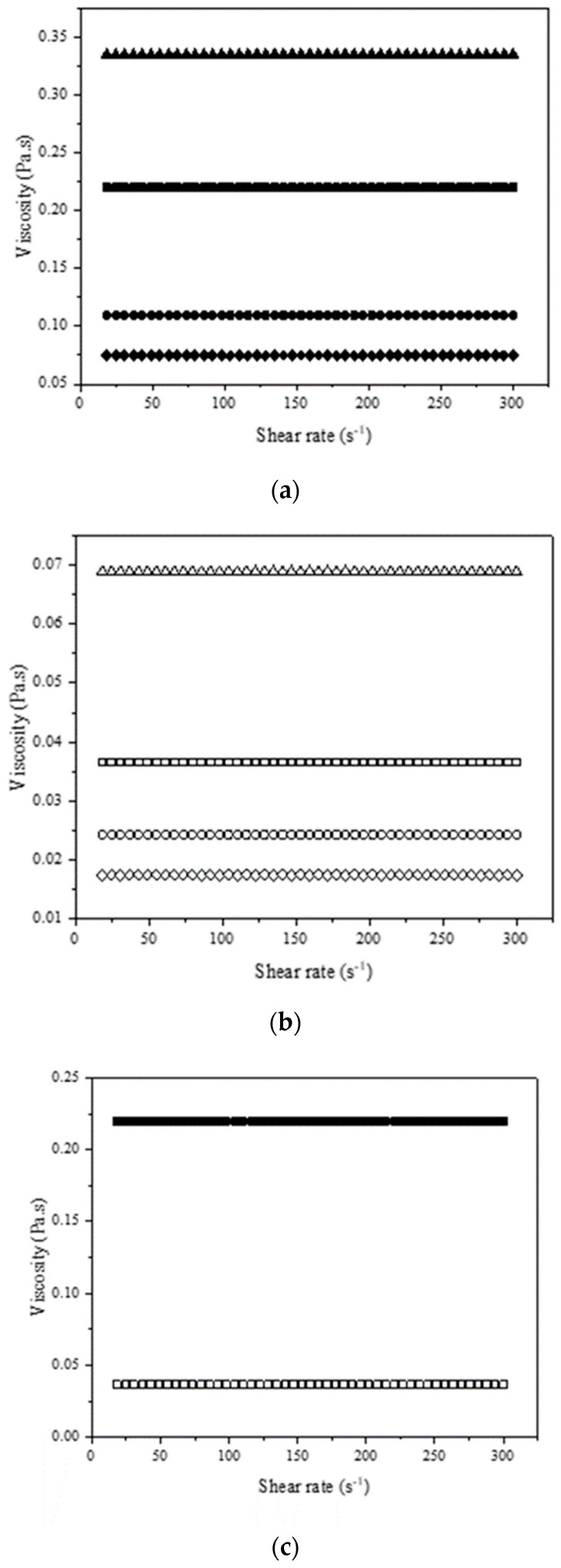
NADESs viscosity profile graph. (**a**) Solid symbols represent NADESs without the addition of water (■ lactic acid:glycine; ● lactic acid:ammonium acetate; ▲ lactic acid:sodium acetate; ♦ lactic acid:choline chloride); (**b**) hollowed out symbols represent NADESs with the addition of water (□ lactic acid:glycine; ○ lactic acid:ammonium acetate; Δ lactic acid:sodium acetate; ◊ lactic acid:choline chloride). (**c**) Comparison of NADESs behavior with and without the addition of water (lactic acid:glycine ■; □).

Viscosity data are relevant for the design stage of industrial processes, fluid flow systems, and in the definition of suitable applications of NADESs, as well as being useful for selecting the optimal relationship of hydrogen bond donors and acceptors [59]. The result of the rheological behavior of the NADESs and a comparison with the lactic acid:glycine mixture at 40 °C are shown in Figure 7 and Figure 8. Figures S9 and S10 (Supplementary Materials) show comparative results of all mixtures. In all the tests, the NADESs showed constant viscosity, being classified as Newtonian fluids. All the NADESs prepared with the addition of water had a lower viscosity than those without the addition of water.

NADES viscosity varies according to their composition and water content [15]. The strong hydrogen bond interactions between the NADES components gradually diminished in the presence of water due to the re-establishment of hydrogen bonding in the system [60]. As a result of their ability to form hydrogen bonds and the reduced size of water molecules, they can act as a plasticizer, decreasing the viscosity [15].

The high viscosity of the NADESs can be advantageous for some industrial applications, such as the production of lubricants [61]. If the fluid is viscous, some chemical applications, such as liquid phase reactions and liquid–liquid extraction, may require high pumping energy [59]. The incorporation of water can promote a viscosity reduction of over 70%, reaching 83% in the lactic acid:glycine mixture. The possibility of adding water to the solvent without compromising its characteristics has been shown to be an attractive alternative that can be applied in NADESs.

The differences found in the rheological behavior of the NADESs can also be associated with the use of different components for the preparation of each mixture. Lactic acid, used in all mixtures, contains a carboxylic group in its structure. The NADESs with the highest viscosity was the mixture with glycine, which is the only component also having a carboxylic group. In organic acids, the carboxyl group is responsible for the intermolecular interactions with the other components in a reaction, promoting hydrogen bonds and increasing solvent viscosity [62].

When comparing water (0.0007 Pa·s 40 °C) with the organic solvents frequently used in analytical procedures, such as methanol (0.0005 Pa·s 40 °C) and ethanol (0.0008 Pa·s 40 °C), the NADESs had a high viscosity [63]. Table 1 shows the experimental values of viscosity (Pa·s), density (g mL^−1^), and the fit parameters of the Newtonian model. The R^2^ and the χ^2^ values indicate that Newton’s model was able to describe the NADESs rheological behavior.

Density is an important physical property used in the design and operation of processes [64]. The developed NADESs presented density values on average 15% higher in relation to water (1.004 ± 0.004 g mL^−1^ at 25 °C), which is higher than other solvents commonly used in extraction processes, such as ethanol (0.783 g mL^−1^ 25 °C), methanol (0.787 g mL^−1^ 25 °C), and acetone (0.785 g mL^−1^ 25 °C) [65].

Similar to viscosity, density is related to free volume and the possibility of finding holes of adequate dimensions, which allows for the movement of solvent molecules or ions [16,66].

The NADESs with the lowest density values were those prepared with lactic acid:choline chloride, with or without the addition of water. Different NADESs at 23 °C showed similar behavior [12]. The incorporation of water to the NADESs decreased the density values between 2 and 4%, which is an expected behavior described in the literature for studies with NADESs [2,63].

### 2.2. Extraction Process Applied to Dragon Fruit

#### Ultrasound-Assisted Extraction

Aiming to define the experimental conditions used to conduct the extraction process, preliminary tests with the NADESs under different conditions using tip ultrasound were performed (Table S1). Based on these results, the NADESs without the addition of water were not used in the extraction study due to their high viscosity. Thus, the conditions adopted in the ultrasound to evaluate the extraction were an amplitude of 70, a time of 10 min, a temperature of 40 °C, and a solute:solvent ratio of 1:10 (*m*/*v*).

Due to the turbidity of the extracts during the analysis of the bioactive compounds, which was not reduced even after a separation step by centrifugation, the extracts in the lactic acid:choline chloride mixture could not be quantified by spectrophotometry. The results of the analysis of the bioactive compounds in the extracts obtained from the different NADESs are described in Table 2.

The produced NADESs were able to extract around 42% of the bioactive compounds from dragon fruit pulp when compared to 60% for ethanol. This result may be related to the higher solubility of ethanol and the efficiency of tip ultrasound [67].

However, when compared to other studies evaluating the extraction of bioactive compounds from different parts of dragon fruit using conventional solvents, such as water and ethanol, combined with ultrasound (UE) or conventional extraction methods, our results were superior [68] to others using water as a solvent (121.86 mg/100 g db and 128.30 mg/100 g bs for the conventional method and UE, respectively), as well as in ethanol 70% extraction (19.72 mg/100 g db) [32]. The extraction efficiency using NADESs is directly associated with their polarity, a characteristic that can be changed by adding water to the formulation. 

The combinations used in this work of lactic acid with glycine, ammonium acetate, or sodium acetate were also efficient for the extraction of phenolic compounds in other plant matrices, mainly medicinal plants [20,58,69,70].

Alternative solvents, substitutes for ethanol, and solvents with high toxicity have been the focus of several studies about their use for the extraction of bioactive compounds [71,72,73,74], with promising results. In this sense, NADESs obtained from natural sources, such as choline, amino acids, and organic acids, are favorable alternatives for use. In addition to their advantages regarding biodegradability, low cost, flammability, and toxicity, they can still be used directly in the food industry without the need to eliminate the solvent [72]. With respect to ethanol, as high concentrations are required to improve the solubility of the compounds for extraction, this solvent has to be eliminated from the extract so that it can be used in the development of products. This procedure can compromise the maintenance of the activity of the extracted bioactive compound, as well as its efficiency [7].

As for the DPPH results, the extracts obtained using the NADESs and ethanol were not statistically different. The same behavior was reported in studies with dragon fruit bark extracts using water and ethanol as solvents [75].

## 3. Methods of Analyses

### 3.1. Reagents

The reagents used for the preparation of NADESs, lactic acid (85% purity) and choline chloride (99% purity), were purchased from the company *Êxodo Científica* (Brazil). Glycine (98.5% purity), ammonium acetate (97% purity), and sodium acetate (99% purity) were purchased from Neon (Brazil). All reagents were of analytical grade.

### 3.2. Preparation of NADESs

NADESs were prepared in accordance with previous work [11,20,60]. Before being weighed on an analytical balance (Shimadzu, AY220, Brazil), the solid reagents were dried in a vacuum oven (MA 030/12, Marconi) at 70 °C for 24 h to remove moisture [76]. In order to select the molar ratio and the percentage of water to be added to the NADESs, preliminary tests were conducted (data shown in Supplementary Material). Table 3 illustrates the developed NADESs and their respective molar ratios.

### 3.3. NADESs Characterization

The developed NADESs were characterized according to the analyses below.

#### 3.3.1. Fourier Transform Infrared Spectroscopy (FTIR)

An FTIR analysis was performed using an Agilent Technologies FTIR spectrophotometer (CARY 630 FTIR, Santa Clara, CA, USA) equipped with a ZnSe ATR system with adjustment for 32 scans and a resolution of 4 cm^−1^. The spectral range was 4000 to 650 cm^−1^ in order to investigate the molecular structure of the analyzed compounds.

#### 3.3.2. Raman Spectroscopy

A Raman analysis was performed using a Horiba Modulated Raman spectrometer (model IHR 320, Kyoto, Japan) with a synapse charge-coupled device (CCD) for signal detection. All spectra were obtained with backscattered geometry. The samples were heated with different lasers of 532, 663, and 785nm (2.41 eV), so the best spectra were selected, and the power was kept low (~0.2 mW) in order to allow thermal equilibrium in the cross-section of the sample. The laser was focused on the sample using a 10X objective lens with a focal length f = 10.5 mm and a numerical aperture AN = 0.35.

#### 3.3.3. Rheological Behavior—Viscosity

In order to determine the rheological behavior of the NADESs, a Brookfield rheometer (model R/S plus-SST, Middleboro, MA, USA) using the C-50 cone plate system coupled to a thermostatic bath (Lauda RE 206 ECOLINE) at 40 °C was used. The shear stress and viscosity data were obtained using the CR ramp method (control rate), with a shear ramp from 0.1 to 300 s^−1^. Analyses were performed in triplicate. The results are presented as means and standard deviations.

The acquired experimental information of shear stress versus strain rate was fitted to the Newton model (Equation (1)) using non-linear regression with Origin 8.0 software (OriginLab, Middleboro, MA, USA). The statistical parameters used to evaluate the fit of the model were the coefficient of determination (R²) and chi-square (*χ*^2^).

*τ* = *nγ* (1)

where *τ* = shear stress (Pa); *n* = behavior index (dimensionless); and *γ* = strain rate (s^−1^).

#### 3.3.4. Density

Density was determined using a hydrometer (GEHAKA DSL-900, São Paulo, Brazil) at a temperature of 25 ± 0.2 °C. Determinations were performed in triplicate, and they are expressed in g mL^−1^.

### 3.4. Application of NADESs in the Extraction Process

After the characterization analysis, the NADESs were used for the extraction of phenolic compounds from freeze-dried dragon fruit pulp (preparation and characterization details are shown in Supplementary Materials).

#### Ultrasound-Assisted Extraction Process

Predetermined amounts of freeze-dried dragon fruit pulp and the NADESs (1:10 *m*/*V*) were weighed and put into a 50 mL Erlenmeyer flask, manually homogenized using a glass rod for 30 s, and taken to a tip ultrasound (QSonica, model Q700, Newtown, CT, USA). The extraction process was conducted at 40 °C (Lauda RE 206, Berlin, Germany) for 10 min with an amplitude of 70, an on/off pulse of 30/20 s, a power of 700 W, and a frequency of 20 kHz. Then, the samples were centrifuged at 107× *g* (Excelsa 4, model 280, Fanem Brazil) for 10 min, and the extract was collected [77,78]. The same procedure and experimental conditions were performed with 60% ethanol (*v*/*v*) for comparison purposes. The extracts obtained were analyzed for phenolic compounds and antioxidant activity (DPPH).

### 3.5. Antioxidant Capacity and Bioactive Compounds

#### 3.5.1. DPPH Analysis

The antioxidant capacity of the extracts was determined [79]. An aliquot of 0.1 mL of the extract was added to 3.9 mL of DPPH solution and read in a spectrophotometer at a wavelength of 515 nm. The antioxidant capacity was expressed in g fruit/g DPPH dry basis.

#### 3.5.2. Total Phenolic Compounds

The total phenolic compounds were determined by using 7.5% sodium carbonate and Folin–Ciocalteu reagent. The results are expressed in gallic acid equivalent mg 100 g^−1^ d.b. [80,81].

### 3.6. Statistical Analysis

The experimental determination results were submitted to an analysis of variance (ANOVA), Tukey’s test (*p* ≤ 0.05), and model adjustment using Software^®^ Statistica Version 7.0 [82].

## 4. Conclusions

The developed NADESs showed physical characteristics of high viscosity and density similar to those of other NADESs; their formation was confirmed using spectroscopy techniques. The NADESs without the addition of water were not feasible to be used as solvents in the process of extracting bioactive compounds from dragon fruit due to their high viscosity; however, their industrial application as lubricants can be investigated. The extracts obtained with the NADESs, such as lactic acid:choline chloride:water, were not analyzed due to turbidity problems. The use of NADESs for the extraction of bioactive compounds from dragon fruit showed promising results. The main advantage consists of using extracts directly or in the preparation of food and pharmaceutical and cosmetic products without the need to eliminate the solvent.

## Figures and Tables

**Table 1:** Viscosity, density, and parameters of Newton’s model for NADESs.

NADESs	Without Water				With Water			
	(Pa s)	ρ (g mL^−1^) ^b,^*	R^2^	χ²	(Pa·s)	ρ (g mL^−1^) ^b,^*	R^2^	χ²
LA:glycine	0.220	1.272 ± 0.002 ^a^	0.99942	0.19631	0.037	1.220 ± 0.001 ^e^	0.98648	0.32691
LA:ammonium acetate	0.109	1.208 ± 0.001 ^b^	0.99474	0.40818	0.024	1.179 ± 0.000 ^f^	0.98744	0.21744
LA:sodium acetate	0.335	1.288 ± 0.001 ^c^	0.99966	0.12422	0.069	1.244 ± 0.002 ^g^	0.99885	0.20942
LA:choline chloride	0.075	1.188 ± 0.002 ^d^	0.99042	0.3181	0.017	1.159 ± 0.002 ^h^	0.98501	0.26056

LA—lactic acid; (*) data show the mean of the triplicate ± standard deviation. Different letters in the same column indicate a significant difference at the 5% level of significance.

**Table 2:** Phenolic compounds and antioxidant potential of dragon fruit extracts obtained using NADESs and ethanol.

Solvents	Phenolics (mg/100g d.b.) *	DPPH (g fruit/g DPPH db)
Lactic acid:glycine	193.18 ± 1.26 ^b^	1765.46 ± 47.43 ^a^
Lactic acid:ammonium acetate	186.08 ± 4.70 ^b^	1623.96 ± 78.50 ^a^
Lactic acid:sodium acetate	157.43 ± 5.37 ^c^	1872.55 ± 169.88 ^a^
Ethanol	450.41 ± 1.74 ^a^	1562.45 ± 185.34 ^a^

Data show the mean of the triplicate ± standard deviation. Different letters in the same line indicate a significant difference at the 5% level. * Data expressed in GAE equivalent.

**Table 3:** Molar ratio of the reagents used for the preparation of NADESs.

NADESs			
Hydrogen Donor	Hydrogen Acceptor	Molar	Water (%)
		Ratio	
Lactic acid	Glycine	4:1	-
Lactic acid	Ammonium acetate	3:1	-
Lactic acid	Sodium acetate	3:1	-
Lactic acid	Choline chloride	3:1	-
Lactic acid	Glycine	4:1	24
Lactic acid	Ammonium acetate	3:1	20
Lactic acid	Sodium acetate	3:1	20
Lactic acid	Choline chloride	3:1	20

## Data Availability

Not applicable.

## Supplementary Materials

The following supporting information can be downloaded at: https://www.mdpi.com/article/10.3390/molecules27238310/s1, Figure S1. Spectra (FTIR) of pure components; Figure S2. Spectra (FTIR) of pure components; Figure S3. Spectra (FTIR) of pure components; Figure S4. Spectra (FTIR) of pure components; Figure S5. Raman spectra of pure components; Figure S6. Raman spectra of pure components; Figure S7. Raman spectra of pure components; Figure S8. Raman spectra of pure components; Figure S9. Flow curves for NADES fitted to Newton’s model at 40 °C. Figure S10. Viscosity profile graph for NADES at 40 °C; Table S1. Tests performed for extractions with NADES.

## Sample Availability

Samples of the compounds are available from the authors.
