# Supplementary material for: Elaboration and Characterization of Natural Deep Eutectic Solvents (NADESs): Application in the Extraction of Phenolic Compounds from pitaya
Source: Molecules. 2022 Nov 29;27(23):8310. doi: 10.3390/molecules27238310 (PMC9739405; doi:10.3390/molecules27238310)
Supplement: Supplementary file 1 [file molecules-27-08310-s001.zip › molecules-2002492-supplementary.pdf]

Elaboration and characterization of natural deep eutectic solvents (NADES): application in the extraction of phenolic compounds

Ianê Valente Pires[a], Yasmin Caroline Nóvoa Sakurai[a], Antônio Manoel da Cruz Rodrigues[a], Nelson Rosa Ferreira[a], Sanclayton Geraldo Carneiro Moreira[b], Luiza Helena Meller da Silva[a]*,

[a]*Programa de Pós-Graduação em Ciência e Tecnologia de Alimentos* [Postgraduate Program in Food Science and Technology], Universidade Federal do Pará, Rua Augusto Corrêa S/N, Guamá, Belém, Pará, 66075-900, Brazil.

[b]*Instituto de Ciências Exatas e Naturais* (ICEN), *Universidade Federal do Pará*, Rua Augusto Corrêa S/N, Guamá, Belém, Pará, 66075-900, Brazil.

NADES behavior (Supplementary Figures and Table)

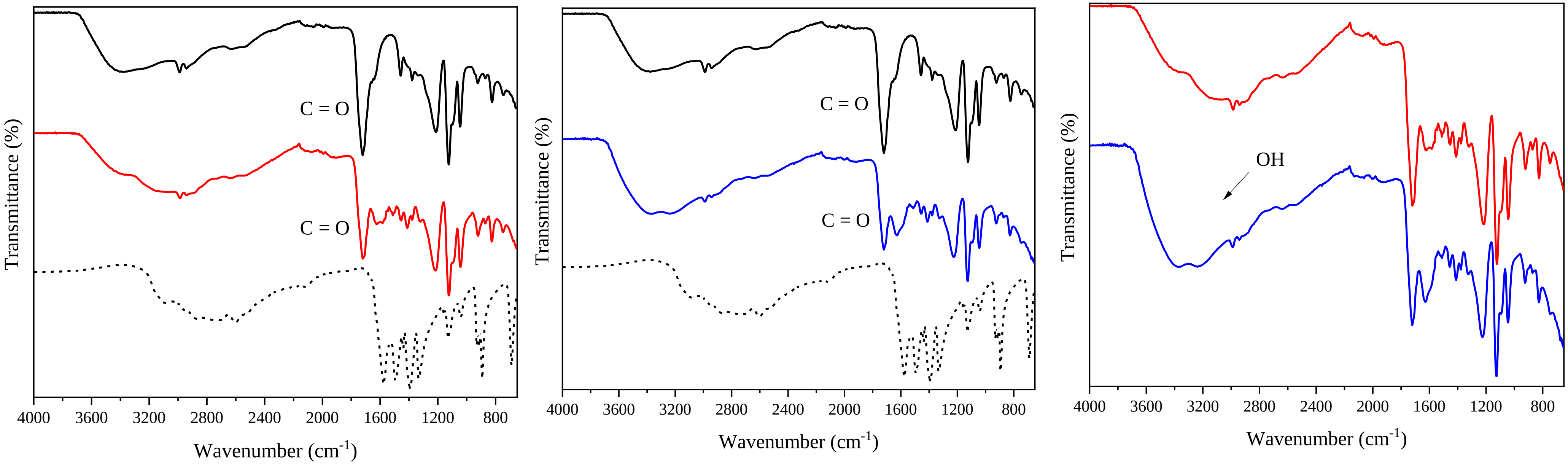

Figure S1. Spectra (FTIR) of pure components. — lactic acid, ---- glycine, — NADES without water; — lactic acid, --- glycine, — NADES with water; comparative behavior of — NADES without water and — NADES with water.

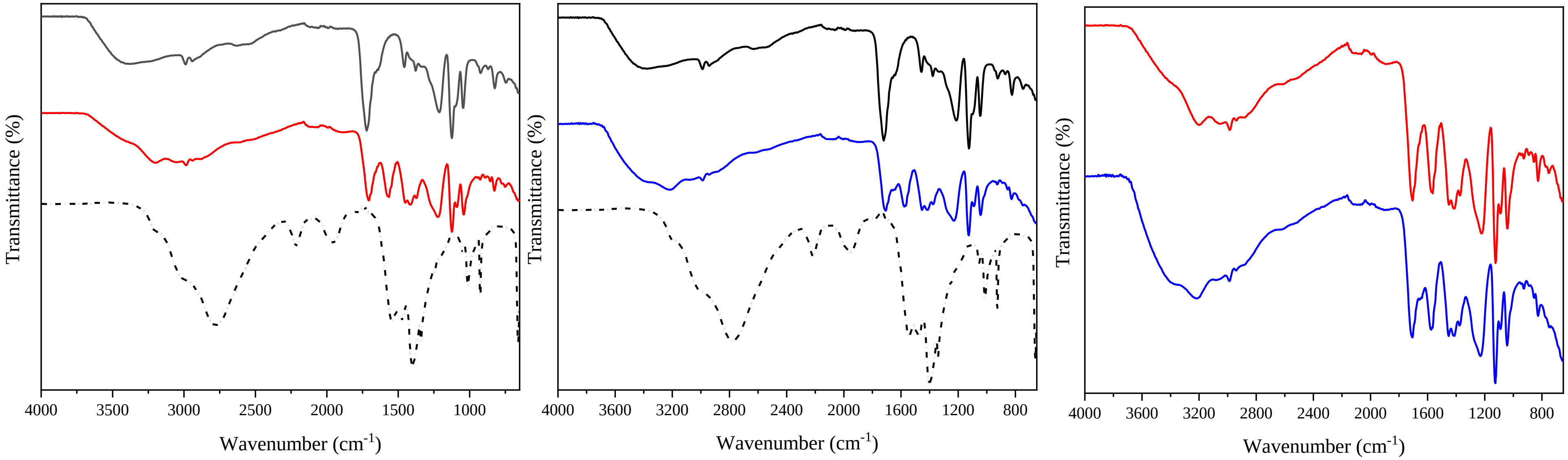

Figure S2. Spectra (FTIR) of pure components. — lactic acid, ---- ammonium acetate, — NADES without water; — lactic acid, --- ammonium acetate, — NADES with water; — NADES without water, — NADES with water.

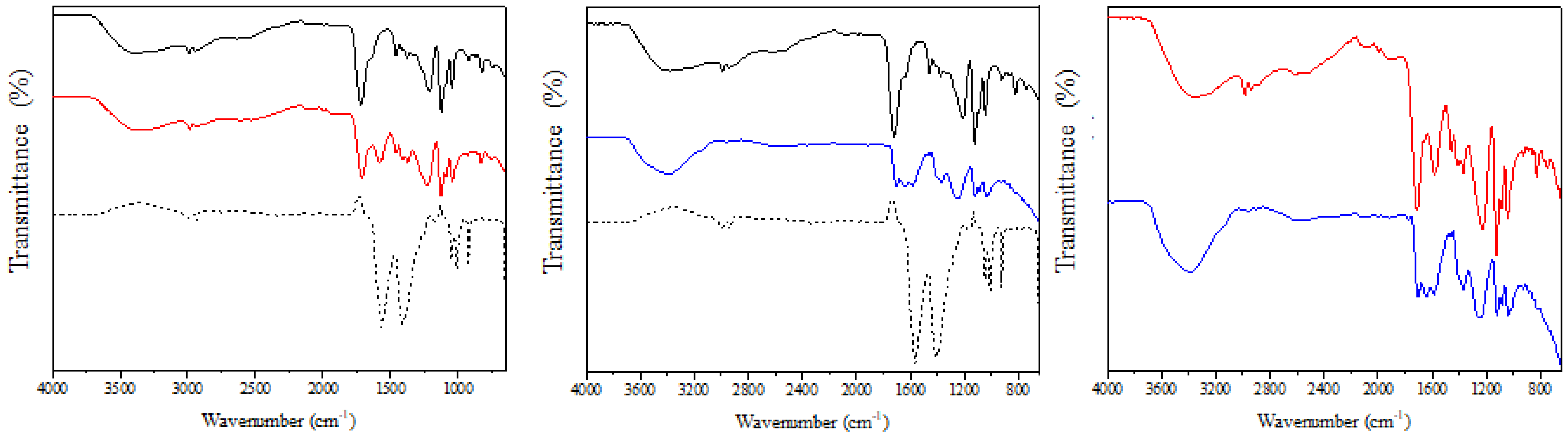

Figure S3. Spectra (FTIR) of pure components. — lactic acid, ---- sodium acetate, — NADES without water; — lactic acid, --- sodium acetate, — NADES with water; — NADES without water, — NADES with water.

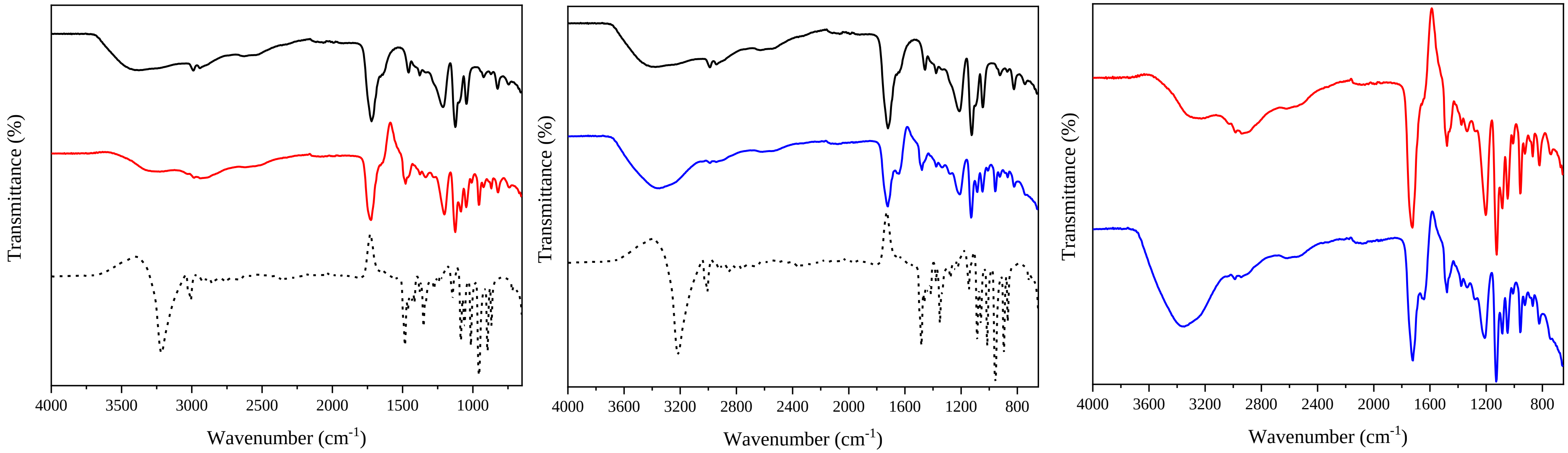

Figure S4. Spectra (FTIR) of pure components. — lactic acid, ---- choline chloride, — NADES without water; — lactic acid, --- choline chloride, — NADES with water; — NADES without water, — NADES with water.

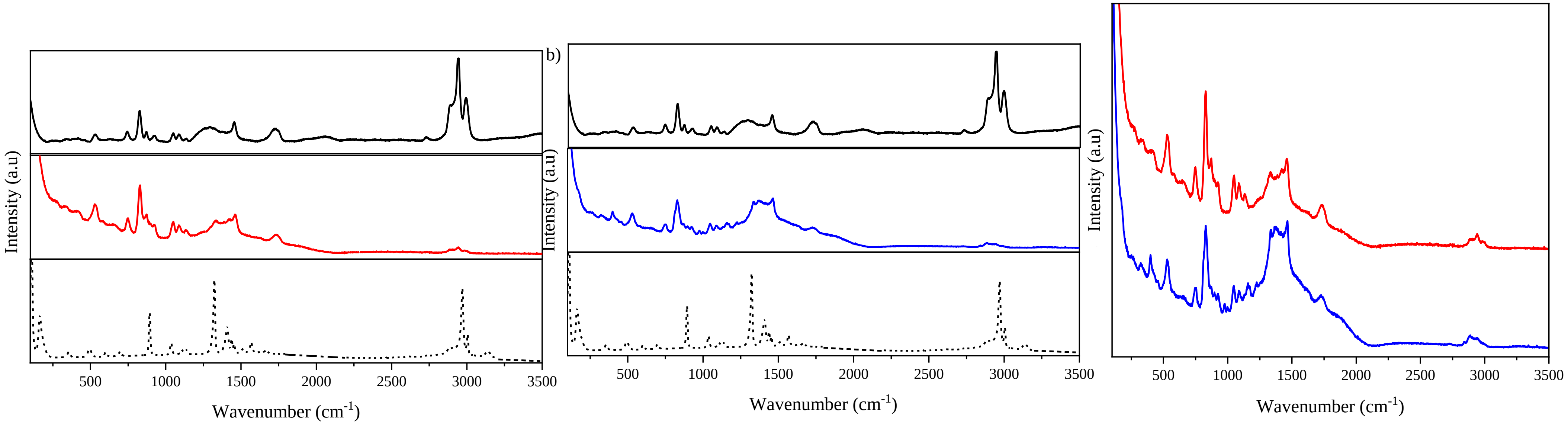

Figure S5. Raman spectra of pure components. — lactic acid, ---- glycine, — NADES without water; — lactic acid, --- glycine, — NADES with water; — NADES without water, — NADES with water.

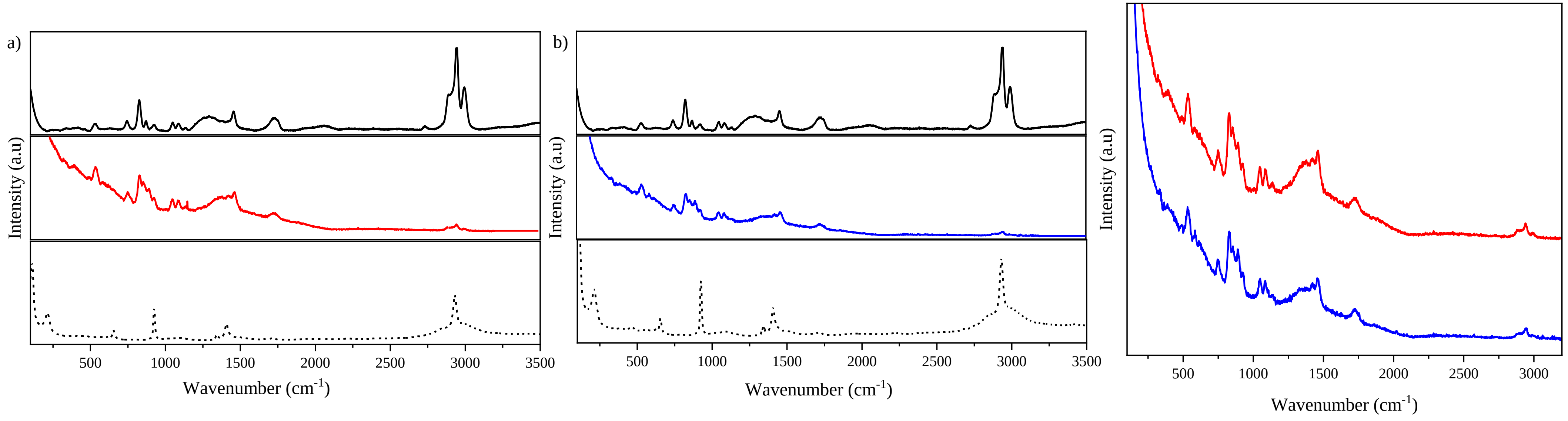

Figure S6. Raman spectra of pure components. — lactic acid, ---- ammonium acetate, — NADES without water; — lactic acid, --- ammonium acetate, — NADES with water; — NADES without water, — NADES with water.

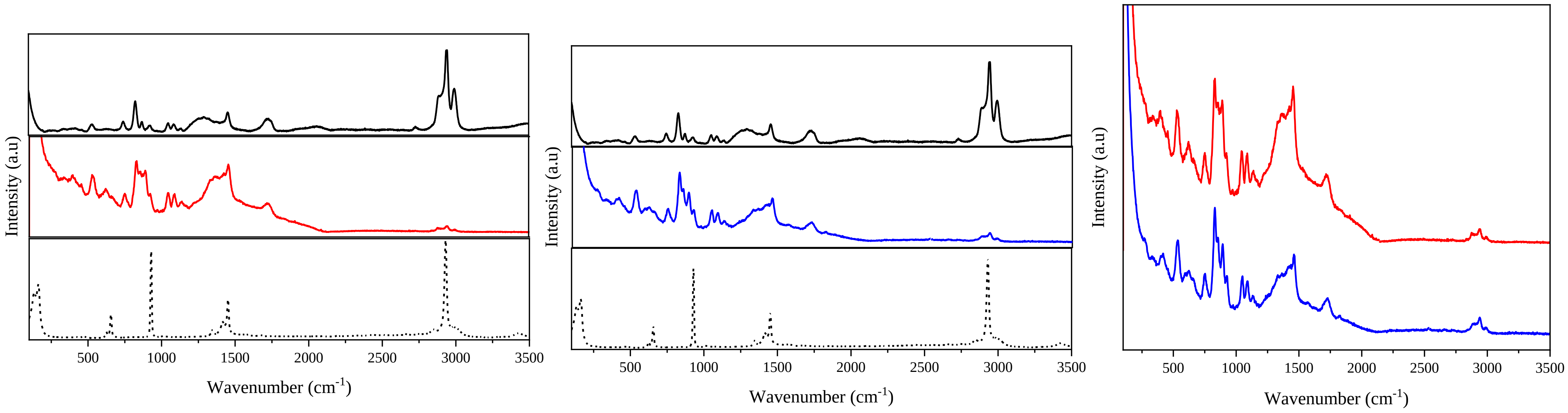

Figure S7. Raman spectra of pure components. — lactic acid, ---- sodium acetate, — NADES without water; — lactic acid, --- sodium acetate, — NADES with water; — NADES without water, — NADES with water.

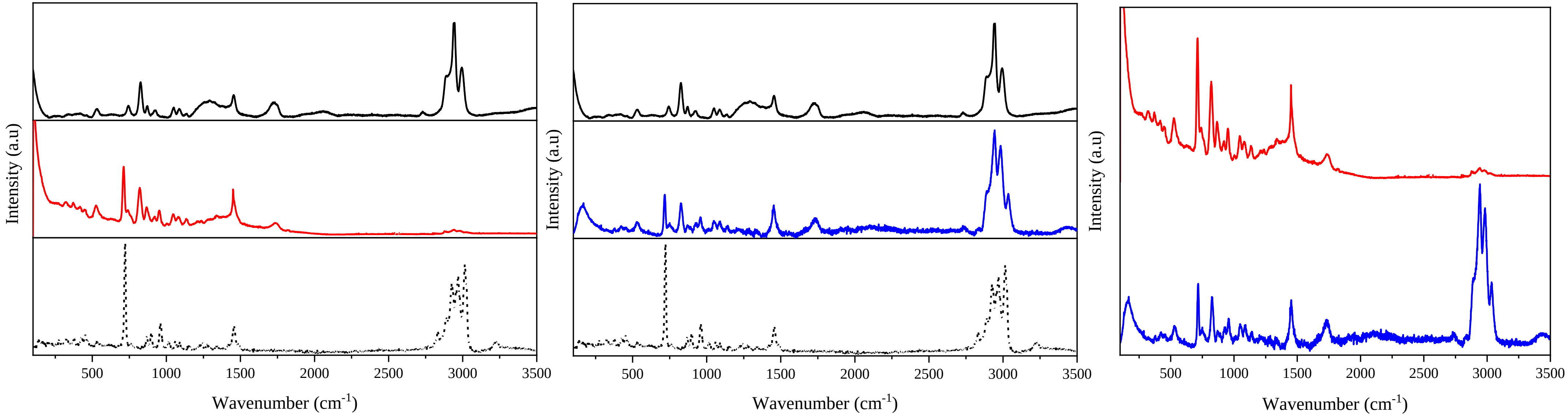

Figure S8. Raman spectra of pure components. — lactic acid, ---- choline chloride, — NADES without water; — lactic acid, --- choline chloride, — NADES with water; — NADES without water, — NADES with water.

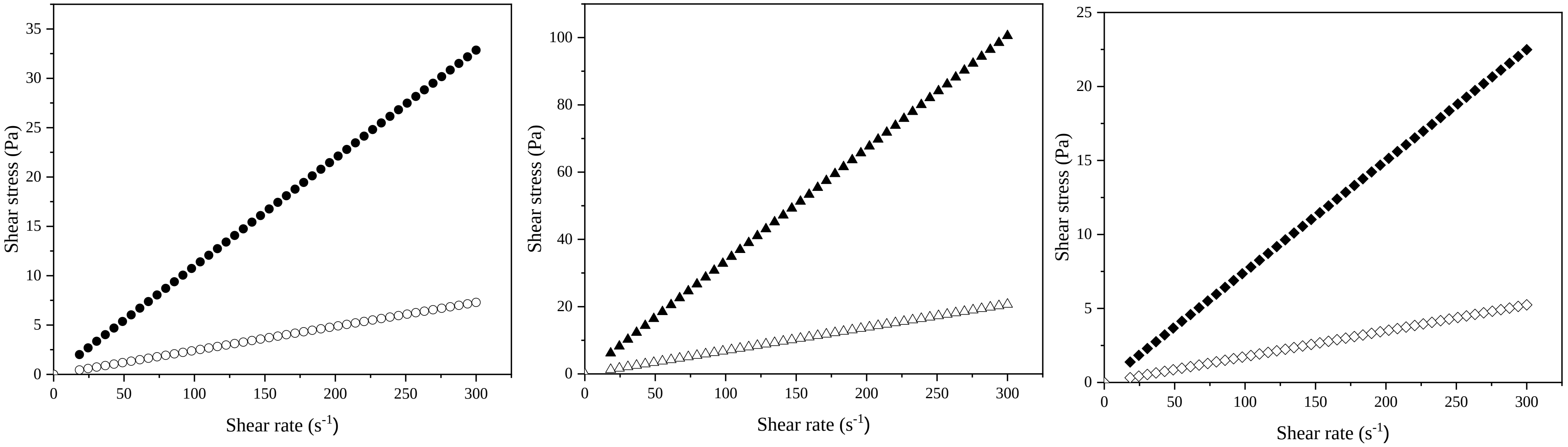

Figure S9. Flow curves for NADES fitted to Newton's model at 40°C. Lactic acid: ammonium acetate ● (without water) ○ (with water); lactic acid: sodium acetate ▲ (without water) Δ (with water); lactic acid:choline chloride ♦ (without water) ◊ (with water).

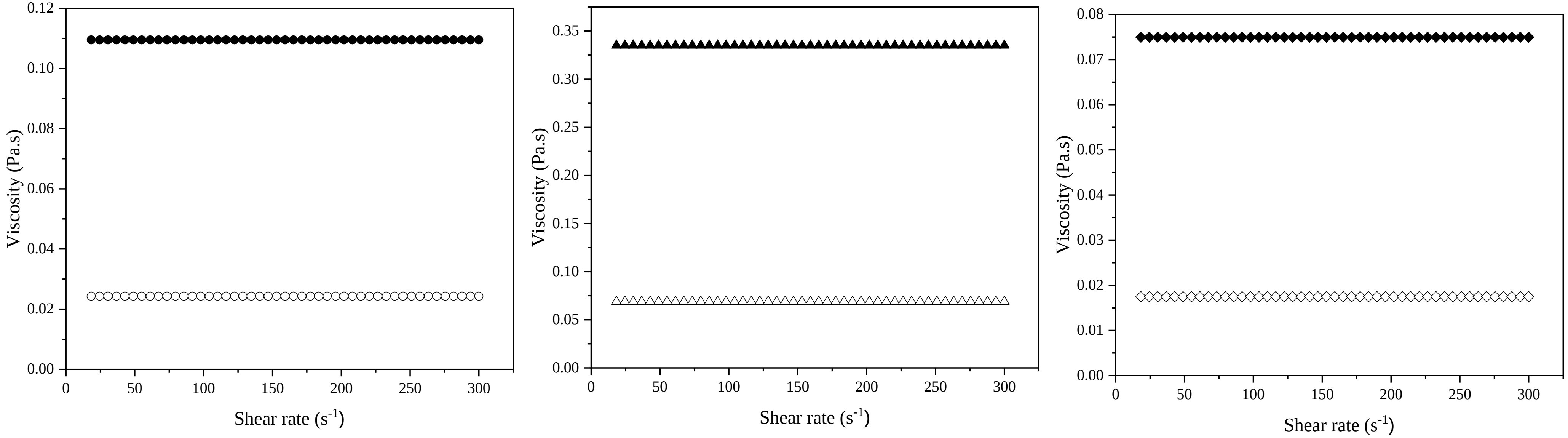

Figure S10. Viscosity profile graph for NADES at 40°C. Lactic acid: ammonium acetate ● (without water) ○ (with water); lactic acid: sodium acetate ▲ (without water) Δ (with water); lactic acid:choline chloride ♦ (without water) ◊ (with water).

Table S1. Tests performed for extractions with NADES.

Tests	NADES	Amplitude	Time (Minutes)	Temperature (°C)	Volume (mL)
1	Without water	60	5	40	20
2	Without water	70	5	40	20
3	With water	70	5	40	20
4	With water	70	10	40	20
